# Enamel Structure Defects in *Kdf1* Missense Mutation Knock-in Mice

**DOI:** 10.3390/biomedicines11020482

**Published:** 2023-02-07

**Authors:** Pei Li, Binghui Zeng, Weihong Xie, Xue Xiao, Ling Lin, Dongsheng Yu, Wei Zhao

**Affiliations:** Guanghua School of Stomatology, Hospital of Stomatology, Guangdong Provincial Key Laboratory of Stomatology, Sun Yat-Sen University, Guangzhou 510055, China

**Keywords:** enamel, hydroxyapatite, micro-computed tomography, transgenic mouse, tooth development, genetics

## Abstract

The *Keratinocyte differentiation factor 1* (*KDF1*) is reported to take part in tooth formation in humans, but the dental phenotype of *Kdf1* mutant mice has not been understood. Additionally, the role of the *KDF1* gene in dental hard tissue development is rarely known. In this study, we constructed a *Kdf1* missense mutation knock-in mouse model through CRISPR/Cas9 gene-editing technology. Enamel samples from wildtypes (WT) and *Kdf1* homozygous mutants (HO) were examined using micro-computed tomography (micro-CT), scanning electron microscopy (SEM), an atomic force microscope (AFM) and Raman microspectroscopy. The results showed that a novel *Kdf1* missense mutation (c. 908G>C, p.R303P) knock-in mice model was constructed successfully. The enamel of HO mice incisors appeared chalky and defective, exposing the rough interior of the inner enamel and dentin. Micro-CT showed that HO mice had lower volume and mineral density in their tooth enamel. In addition, declined thickness was found in the unerupted enamel layer of incisors in the HO mice. Using SEM and AFM, it was found that enamel prisms in HO mice enamel were abnormally and variously shaped with loose decussating crystal arrangement, meanwhile the enamel rods were partially fused and collapsed, accompanied by large gaps. Furthermore, misshapen nanofibrous apatites were disorderly combined with each other. Raman microspectroscopy revealed a compromised degree of order within the crystals in the enamel after the *Kdf1* mutation. To conclude, we identified enamel structure defects in the *Kdf1* missense mutation knock-in mice, which displayed fragmentary appearance, abnormally shaped prism structure, decreased mineral density, altered crystal ordering degree and chemical composition of the enamel layer. This may support the potential role of the *KDF1* gene in the natural development of enamel.

## 1. Introduction

As the outermost layer of the human tooth, enamel is gathered by hard, brittle hydroxyapatite (Ca_10_(PO_4_)_6_(OH)^2^) (HAP) with strategically placed flexible organics such as enamelin, ameloblastin and tuftelin [1,2]. The regular decussating arrangement and compact bonding of mineral crystals, which are mildly misoriented with adjoining ones, reflect a sophisticated hierarchical microstructure of enamel [3,4,5]. Thus, enamel is a natural design combining high stiffness and extreme fracture toughness with outstanding success. As it should be, the processes of enamel formation are highly regulated at the molecular level with essential genes such as *enamelin (ENAM), kallikrein 4 (KLK4), matrix metalloproteinase 20 (MMP20)* and others [6]. Mutations in these genes can alter regular molecular pathways, leading to various enamel phenotypes. For example, variants in ENAM affected the secretion of enamelin by ameloblasts both in human and mice models, thus resulting in deficient enamel and declined wear resistance of the teeth [7,8,9]. In *Klk4* knockout mice, terminal extracellular degradation was disabled in a maturation stage and consequently discolored the enamel layer [10,11,12,13]. The developing tooth-specific proteinase enamelysin is encoded by the *MMP20* gene. Patients with *MMP20* missense mutation caused the autosomal-recessive hypomaturation type of amelogenesis imperfecta; the *Mmp20* null mice model showed a mineral composition change and therefore enamel was easily detached from the dentin [14,15]. Thus, certain gene mutations can lead to enamel abnormalities and poor function but many of them are poorly understood.

*KDF1* is located on chromosome 1p36. Patients with 1p36 deletion syndrome presented abnormal tooth morphology and delayed tooth eruption [16,17]. In the *Kdf1* knockout mouse model, *Kdf1* was found to be expressed in epidermal cells from the earliest stages of epidermis creation through adulthood, regulating proliferation as well as differentiation in epidermal progenitor cells and having an important influence on tissue morphogenesis and skin barrier integrity [18]. Unfortunately, the *Kdf1* knockout mice die shortly after birth due to epidermal barrier deficiency, so the dental phenotype was not observed. Patients with the *KDF1* missense variant (c.753C>A, p.F251L) displayed an autosomal dominant form of ectodermal dysplasia in their families, including absent teeth, thin eyebrows, nail changes and decreased sweating [19,20]. In our earlier research [21], the missense mutation of *KDF1* (c. 908G>C, p.R303P) proved to be pathogenic in non-syndromic tooth agenesis patients through whole exome sequencing and bioinformatics analysis. We further found that in the developing tooth germ of mouse embryos, *Kdf1* was strongly expressed in the dental epithelium at the bud stage and the cap stage and mainly expressed in the inner enamel epithelium at the bell stage. Similarly, Pan et al. [22] identified a novel de novo missense mutation (c.911T>A, p.I304N) in the *KDF1* gene in a Chinese patient with severe non-syndromic anodontia. In vitro, functional studies showed altered mRNA and protein expression levels of R303P, F251L and I304N of the mutant *KDF1* [22]. However, it was possible that this mutation was partially linked to the tooth phenotype instead of being causative. Additionally, *KDF1* mutations to date contain multiple variant forms including missense mutation, copy number loss and deletion of the gene. To further evaluate the potentially causal link between the variant and malformed tooth, as well as help delineate the phenotype of the *Kdf1* mutant in mice model and pathogenic mechanism, we report here for the first time the dental phenotype of mice model with *Kdf1* gene missense mutation (c. 908G>C, p.R303P), which turn out to show enamel structure defects; additionally, we analyzed their changes of microstructure, thickness along with mineral density and the crystal properties of the enamel. Based on previous studies and our findings, *KDF1* may be involved in enamel formation and maturation.

## 2. Materials and Methods

### 2.1. Generation of Kdf1 Knock-in Mice with a Missense Mutation

Mice were obtained from a breeding colony maintained in a C57BL/6 background. *Kdf1* knock-in mice with a pathogenic mutation (R303P: replacement of Arg303 with Pro) were generated using the CRISPR/Cas9 system. The plasmid vector pX330 was obtained from Feng Zhang Lab. Single-guide RNA (sgRNA) and Cas9 were expressed in the vector. The human and mouse *KDF1* gene sequences were downloaded from NCBI (https://www.ncbi.nlm.nih.gov/gene/, accessed on 19 Febuary 2019). The c.G908 mutation position in the human *KDF1* gene is located at the same position (c.G908) in the mouse *Kdf1* gene. The guide RNA design tool (https://zlab.bio/guide-design-resources, accessed on 19 Febuary 2019) was used to design sgRNA in silico. The target sequence and protospacer adjacent motif (PAM) sequence was CCGTATTAGTACCCGCAAAAGCC (The PAM sequence is shown in bold letters). In order to avoid secondary cleavage, since the point mutation of R303P (CGT>CCT) will introduce a new CCT (reverse complementary NGG), a synonymous mutation of I304 (ATT>ATC) was generated. The sgRNA (25 ng/μL) and Cas9 mRNA (25 ng/μL) were injected into C57BL/6 mouse zygotes in the presence of 91 bp single-stranded oligonucleotides (ssODN) (100 ng/μL) using a microinjection system under standard conditions. The ssODN sequence was: CAGGACTACCACCTGGATGAGCAAGACGCCGAGGGCCGCCTGGTGCGGGGCATCATCCcTATcAGTACCCGCAAAAGCCGCTCCCGCCCACAGACCTCCGAGGGGCGCTCAGCCCGCTCT (the mutation site is shown as a lowercase letter). We constructed gRNA and cas-9 homologous recombination plasmids for the target gene. The zygotes were cultured in a culture medium at 37 °C in 5% CO_2_ up to the two-cell embryo stage and then the embryos were transferred into the oviducts of recipient mice. After the founder mice were born, we clipped their tails and extracted DNA. PCR and sequencing were performed to identify the three positive founders. Tail genomic DNA was amplified using a specific primer set: forward 5′-AGCACCTTCACCAACAGCC-3′ and reverse 5′-AAAGCCCAATCCTTCTCCT-3′. F1 with the heterozygous c. 908G>C mutation was obtained by crossing between mutant founders (F0) and C57BL/6J parents. F2 offsprings with the homozygous c. 908G>C mutation were generated from the crossing between heterozygous F1 offsprings. The genotyping of the homozygous mice we generated was confirmed through PCR analysis and Sanger sequencing of genomic DNA extracted from mouse tail clips to detect the targeted allele. All animal procedures adhered to (Animal Research: Reporting of In Vivo Experiments) guidelines for animal research and were approved by the Experimental Animal Ethics Committee of Sun Yat-sen University (Approval number: SYSUIACUC-2020-000908).

### 2.2. Sample Preparations and Processing

**Figure 1:**
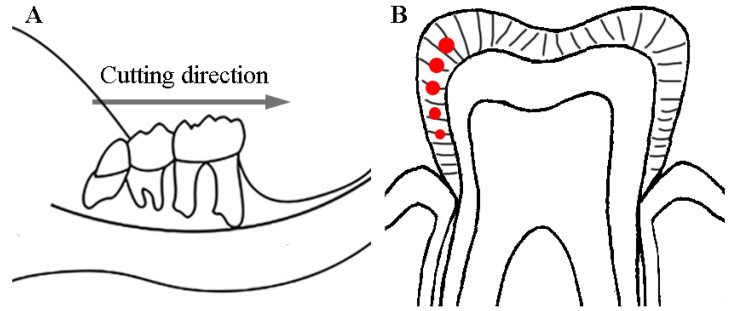
The sketch of the sample preparation method: (**A**) The cutting direction went along the mesial and distal direction of the teeth. (**B**) The sectional drawing shows the axial surface of the tooth; the red dots indicate that the detection area was in the middle layer of enamel in the M1 molars.

Twenty-five WT mice (3 months old, weight 23.2 ± 2.71 g) for the WT group and twenty-five HO mice (3 months old, weight 22.6 ± 3.67 g) for the HO group were sacrificed after anesthesia. Their mandibles were fixed in 2.5% glutaraldehyde for 1 h at 4 °C. After rinsing with distilled water, twenty mandibles were dehydrated from each group and made into slices for section views in the tests and the rest were prepared for micro-CT scans. These samples for the section were first embedded in epoxy resin. A roulette cutter (YWQ-50S, WEIYEE, Guangzhou, China) was used to cut along mesiodistal directions. A grinding and polishing machine (UNIPOL-1502, Kejing, Hunan, China) along with polishing liquid and mesh sandpapers were used to narrow every slice to a thickness of 0.8–1 mm. Figure 1A shows the direction of the prepared slices within the anatomical region. The red dots (Figure 1B) mark the middle layer enamel of M1 molars as test sites in SEM, AFM and Raman spectroscopy analysis.

### 2.3. X-ray Microtomography

Six hemimandibles from WT and HO mice with 3 mice per group were used for micro-CT (SCANCO μCT, Zurich, Switzerland) scans, respectively. These mandibles were wrapped in normal saline-soaked gauze and frozen at −20 °C until use. After the mandibles were thawed at room temperature, the specimens were scanned by micro-CT operated at 70 kV with a source current of 200 µA and an aluminium filter to reduce beam hardening. CT images were calibrated using a five-point standard of hydroxyapatite mineral of known densities (1.13, 1.2, 1.26, 1.4 and 1.65 g/cm^3^). The same setup and parameters were used for the phantoms and the tooth specimens. Regions of interest (ROIs) were drawn around the enamel of the M1 and erupted incisors for mineral densities, respectively. CT values were converted into mineral density values (gHAp cm^−3^) according to a linear calibration curve based on the corresponding CT values of each phantom (linear regression, R^2^ > 0.9982) [23,24]. As for the observation of enamel thickness, ROIs were drawn around the enamel of the unerupted incisors. The contrast positions were chosen at the first molar distal root, second molar mesial roots and third molar roots in the transverse microtomographic views [25,26,27]. To measure the enamel volume and area of the six hemimandibles, we applied the same threshold cutoff to ensure that only highly mineralized structures are visible [13]. The 3D reconstruction of hemimandibles was processed by mimics 21.0. ROIs were drawn around the M1 molars when proceeding with false-color observation. The volumes and area statistics along with false-colored procedures of the resulting scans were conducted by ImageJ software 13.0.6 (https://imagej.nih.gov/, accessed on 12 March 2021). Average volumes and areas were statistically compared.

### 2.4. Scanning Electron Microscopy

#### 2.4.1. Visualization of Prism Structures

Five defleshed slices per group were treated with acid (37% phosphoric acid for 20 s) and ultrasonically cleaned [28]. Samples were later fixed and coated with a 10 nm layer of gold using graded ethanol (50%, 70%, 95%, 100% ethanol) dehydration. Each sample was imaged by SEM with identical imaging conditions at 20 kV (ZEISS, Oberkochen, Germany).

#### 2.4.2. Calcium-to-Phosphorus Ratio

Three slices (not treated with acid) from each group were tested for calcium-to-phosphorus ratios using energy dispersive X-ray spectroscopy (ZEISS, Oberkochen, Germany). Three square areas of 20 × 20 μm in the middle layer of enamel were analyzed from each sample separately for >5000 cts.

### 2.5. Raman Microspectroscopy

Five samples from WT and HO mice were used to assess the structural and compositional differences, respectively. A Confocal Raman microscope (RENISHAW, London, England) emitting 785 nm wavelength light was used to detect a 3 µm spot size and focus on the middle layer of enamel in M1 molar through a Leica 50× long-working distance objective with NA (numeric aperture) = 0.5. Spatial resolution was 5 μm, detector integration time was 10 s and each final curve resulted from 30 averaged spectra. A wide-range grating in the range of 300–2000 cm^−1^ was used for the Raman measurements. The raw spectra were subjected to a cosmic spike removal algorithm (sensitivity: 3, spikes width: 7). Subsequently, the baseline shifts due to tissue autofluorescence were corrected by fitting a twelve-point third-order polynomial to the spectra. Finally, the average Raman spectra were normalized in relation to the peak of the phosphate band to remove the differences in the total Raman intensity due to the physical and optical factors by Scikit-learn modulus in the python 3.7 platform. Normalized Raman bands were used to assess the carbonate/phosphate ratio by dividing and calculating integrated areas under the curve after the Gaussian and Lorentz fit. The peak position of the phosphate band at 960 cm^−1^ and carbonate band at 1070 cm^−1^ were determined using Origin 2018 software (Origin Lab, Northampton, MA, USA).

### 2.6. Atomic Force Microscope Analysis

Three specimens from each group were selected randomly for the investigation of AFM (Bruker, Karlsruhe, Germany) after acid-etched treatment using a V-shaped cantilever probe and contact mode. In accordance with the pre-experiment records and the literature, the MuLT1-190-1 silicon probe was chosen. The radius of curvature of the probe was 8 nm, and the applied peak force was 2.5 μN. The elastic constant of the cantilever arm was 1.0 N/m. We set up three parallel test points with 100 μm intervals between each point in the middle layer of enamel from each sample. Areas of 5 × 5 μm squares were scanned at the rate of 2 μm to determine the topography and force-displacement curve. All tests were completed at 25 °C and 40% relative humidity. NanoScope Analysis software was used to analyze the approaching curve and calculate Young’s modulus of the samples according to the elastic contact model (Hertzian (Spherical)). After automatic baseline correction, the corresponding Young’s modulus of each point is obtained [29]. The average modulus of the three test points was taken as Young’s modulus of the area. Nanoscope analysis software was also set to flatten, construct 3D images and obtain roughness parameters (average roughness (Ra), mean root squared surface roughness (Rq) and maximum surface roughness (Rmax)) as well.

### 2.7. Statistics

Statistical analysis was conducted by GraphPad Prism 8.3 ( GraphPad Software, San Diego, CA, USA). The measurement data were presented as means ± standard deviation (SD). The Shapiro–Wilk test was used for the normality test and the F-test was used for the homogeneity of variance. If the data conformed to a normal distribution and the variance was uniform, the Student’s *t*-test was adopted. The Mann–Whitney test was used for those whose normality did not meet the conditions and Welch’s *t*-test was used for those whose variance did not meet the conditions. *p* values < 0.05 were considered statistically significant.

## 3. Results

In our present study, we compared the teeth of *Kdf1* mutant and WT mice from macroscale to nanoscale to explore the differences in enamel formation characteristics. Furthermore, microstructures, thickness and mineral density and crystal properties were investigated in detail.

### 3.1. Generation of Kdf1 Knock-in Mice with a Pathogenic Mutation (R303P)

**Figure 2:**
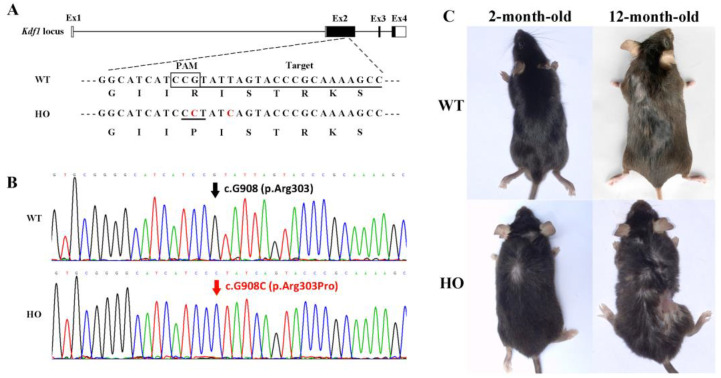
Generation of *Kdf1* R303P knock-in mice using CRISPR/Cas9: (**A**) The original sequence in the *Kdf1* locus was changed into a knock-in allele c. 908G>C, leading to a pathogenic missense mutation. Four exons (Ex1–4) were shown in boxes. The sgRNA target sequence and PAM sequence were marked with an underline and a square, respectively. In the knock-in allele, the mutated nucleotide is highlighted in red. The base sequences of the wild-type allele and the knock-in allele, respectively, are compared to the amino acid sequences of the original KDF1 protein and the altered protein. (**B**) Sequencing results showed that F2 homozygous mice with c. 908G>C were successfully generated. (**C**) Pictures of 2-month-old and 12-month-old WT and HO mice were shown. The 12-month-old HO mouse showed severe skin damage.

We created a *Kdf1* knock-in mouse strain bearing R303P using the CRISPR/Cas9 technology and assessed the pathogenic consequences of missense mutations experimentally. The 91 bp single-stranded DNA (ssDNA) with the pathogenic mutation, as well as a target sequence of sgRNA in the vicinity of the mutation site, are shown in Figure 2A. F1 heterozygous mice and F2 homozygous mice were obtained as described. The genotype of homozygous F2 confirmed by sequencing utilizing the PCR-amplified products is presented in Figure 2B. We observed some general characterizations of the HO mice. All the gerontic ones (12-month-old) showed abnormal skin damage in different levels (Figure 2C).

### 3.2. The Appearance of Enamel Surface in Kdf1 Mutant Mice

**Figure 3:**
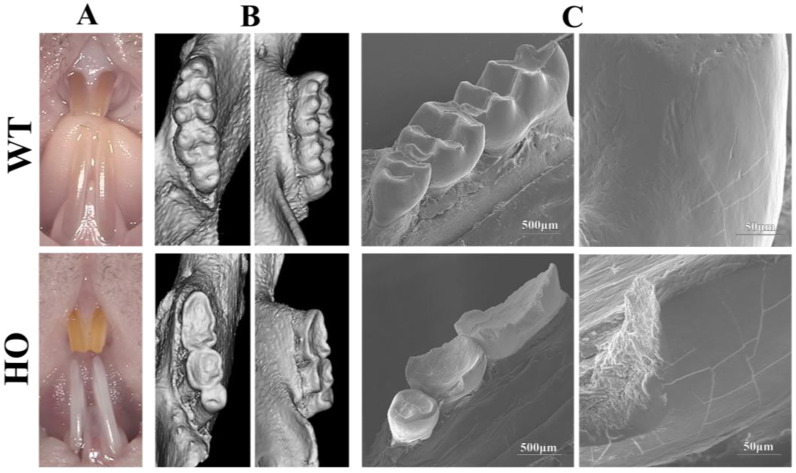
Teeth from HO mice showed severe enamel structure defects: (**A**) incisors of the HO mice looked chalky and thin compared to normal incisors; (**B**) representative CT reconstruction on the occlusal and axial plane of defleshed mandibles demonstrated complete cusps in WT mice molars but a concave surface in HO mice molars; (**C**) SEM presented that HO mice teeth appeared rough and incomplete.

Teeth eruption of four incisors and twelve molars was completed after 4 weeks in both the WT and HO mice. However, when we observed their teeth closely, HO mice had distinct enamel phenotypes. The incisors of WT mice displayed a polished and transparent enamel surface, whereas HO mice incisors appeared abnormally chalky and slender (Figure 3A). When it comes to molars, we presented the left sides of mandibles for reference by micro-CT (Figure 3B) and SEM (Figure 3C) views since symmetrical characteristics can be observed on both sides. Complete cusps can be seen on all molars of WT mice and the enamel was mostly smooth; however, the shape of molars in the HO mice was incomplete. There were no visible pits or fissures. Occlusal surfaces appeared dented with sharp edges around them. Part of the enamel layer in the axial planes was missing, exposing the rough interior of the inner enamel and dentin.

### 3.3. Enamel Prism Structure and Mechanical Properties in Kdf1 Mutant Mice

**Figure 4:**
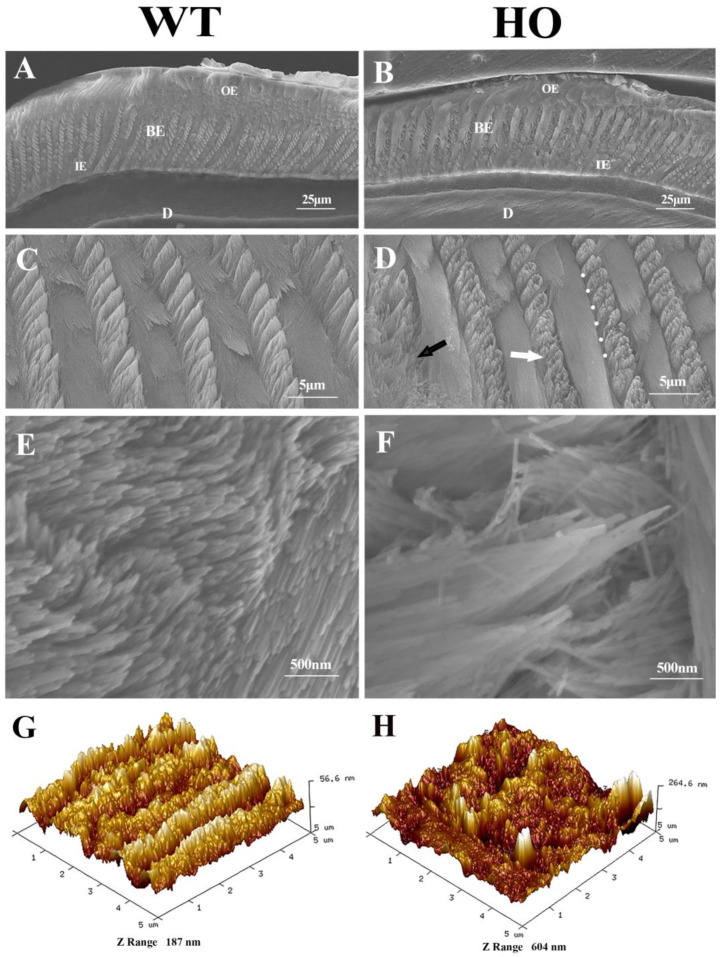
SEM image and topography of the enamel prismatic structure: (**A**,**B**) Cross-section of axial enamel layer in the first molars. OE: Outer enamel; BE: Body enamel; IE: Inner enamel; D: Dentin. (**C**) Typical columnar structure of enamel prisms in WT mice enamel. (**D**) HO mice enamel showed distorted and sparse areas (white arrow) composed of cluttered nanofibrous apatites (black arrow), with wider interprismatic gaps (dots marked). (**E**) Regular packing crystals in WT mice enamel. (**F**) Crystallites lined up in disorder with large spaces between prisms in HO mice enamel. (**G**,**H**) AFM showed regular decussation in WT mice. HO mice enamel appeared with collapsed and fused prisms and large cracks through interprisms.

All electron micrographs of HO mice teeth slices showed the same prism structure. Figure 4 gives representative examples of the SEM morphologies of the sections from WT and HO mice samples. WT mice molars displayed a typical decussating and well-organized enamel prism structure, which was classically described in many studies (Figure 4A,C,E). In the HO mice molars, however, their enamel rods mostly maintained comparable arrangements of single-layered rows and cross orientations but were variously and oddly shaped. Some of the prisms looked like distorted bunches of crystals and they collapsed at the tip of the cone (Figure 4D, white arrow). Others could not even be recognized as a typical decussating pattern (Figure 4D, black arrow). Only a few parts showed an organized enamel prism structure. The organic sheet at the interprisms was dissolved by etching acid, and larger gaps were found between loosely gathered rods in the HO mice enamel (Figure 4D, dots marked). Beyond that, it seemed that nanofibrous apatite in the HO enamel did not gather in the same direction at the tip as that in the WT enamel (Figure 4F).

Confirmed by SEM test, AFM images revealed regular decussating patterns with a relatively flat surface in WT mice enamel. With most prisms wrecked and collapsed, the HO mice enamel appeared rather rough in topography, reflecting the ragged edges around prisms composed of anisotropic crystals. Over-dissolved interprisms lead to large cracks, making the exposed top of the prisms resemble blunt cones, which are distinguished by their altitude height (Figure 4G,H). The image average roughness parameters Ra, Rz and Rmax, respectively, showed significant differences between the two groups (*p* < 0.01). In addition, there was a significant decrease in Young’s modulus calculated from the force-displacement curve in the HO group compared to the WT group (Table 1, *p* < 0.05).

### 3.4. The Mineral Density and Thickness of Enamel Layer in Kdf1 Mutant Mice

**Figure 5:**
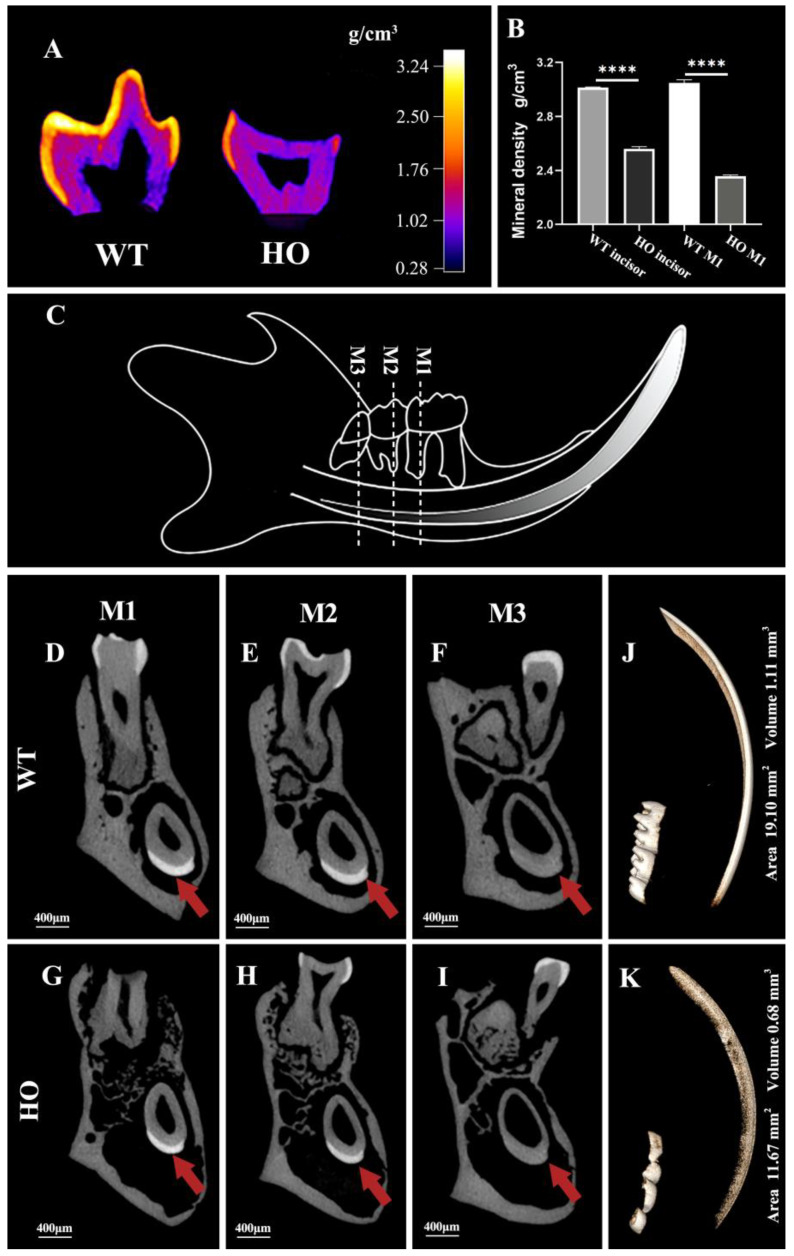
Micro-CT analysis of enamel from WT and HO mice: (**A**) False color plots showed that the enamel of the WT mice molar was generally thick and mineralized gradually, with the surface enamel being the most mineralized and the EDJ the least, generating a well-demarcated margin; the enamel of HO mice was thinnish and more radiolucent and did not show a distinct boundary. (**B**) There was a significant difference in the enamel mineral densities between the WT and HO mice incisors and molars: **** *p* < 0.0001. Error bars denote mean ± SEM. (**C**) The diagram with dotted lines points out the chosen positions of the transverse section of the mandible in mice. (**D**–**F**) In the WT mice, the enamel layer mostly covered the crown of the molars and the labial aspect of the incisor, where it slowly decreased in thickness and radio-opaque from tip to apical. (**G**–**I**) In the HO mice, the enamel on the molars was barely visible and that on the incisors was flimsy and dim (arrowheads). (**J**,**K**) Representative 3D micro-CT reconstruction of the hemimandibles was presented with a threshold cutoff at which only highly mineralized structures were seen. The volume and area of the high threshold region of the WT sample were 19.10 mm^3^ and 1.11 mm^2^. The volume and area of the high threshold region of the HO sample were 11.67 mm^3^ and 0.68 mm^2^.

The false color images of molars by micro-CT demonstrated that enamel from WT mice varied in its mineral density with a general trend of gradual decrease from the surface to the enamel–dentine junction (EDJ) (Figure 5A). There is a clear boundary between enamel and dentin. The enamel layer in HO mice was absent on the occlusal plane and thinner on the axial plane. The average mineral density of enamel in M1 of HO mice (2.36 ± 0.008 g/cm^3^) was lower than that of WT mice (3.05 ± 0.023 g/cm^3^) (*p* < 0.0001). Therefore, the mineral density of enamel in HO mice is not as high as that in WT mice and the EDJ projection is not as obvious as that in WT mice. As for incisor enamel, its shape is more regular than that of molars, and the regionally obtained CT values are stable enough to serve as a reference for mineral density calculation. The mineral density calibration curve was calculated by linear regression based on the grey scale values obtained from the mineral reference phantoms (R^2^ > 0.99). The average enamel mineral density of HO mice incisors is 2.56 ± 0.016 g/cm^3^ and that of WT mice incisors is 3.02 ± 0.002 g/cm^3^. The graph shows that there was a significant difference in the enamel mineral densities between the two groups (*p* < 0.0001) (Figure 5B).

X-ray microtomographic images clearly revealed the corresponding transverse section of the enamel layer of the molars and the unerupted part of the incisors (Figure 5D–I). In the HO mice, comparable scans showed the thinner and less bright layer formed over the dentin (Figure 5G–I, arrowheads), which was barely distinguishable at the apical area (Figure 5I). In the region examined, the lower radio-opaque appearance indicates the decreasing formation and mineralization of the enamel. The enamel layer of the three molars and the incisor in the WT hemimandible were evidently above the threshold. Dentin and bone were below the threshold (Figure 5J). In the molar and incisor regions of the HO mice, there was an obvious lack of integrity in the above-the-threshold region (Figure 5K). We further evaluated the volume and area statistics of the above-the-threshold region. There was a significant drop in the HO mice enamel area (*p* < 0.001) and volume (*p* < 0.05) relative to the WT group.

### 3.5. Mineral Composition and Crystal Properties in Kdf1 Mutant Mice Enamel

**Figure 6:**
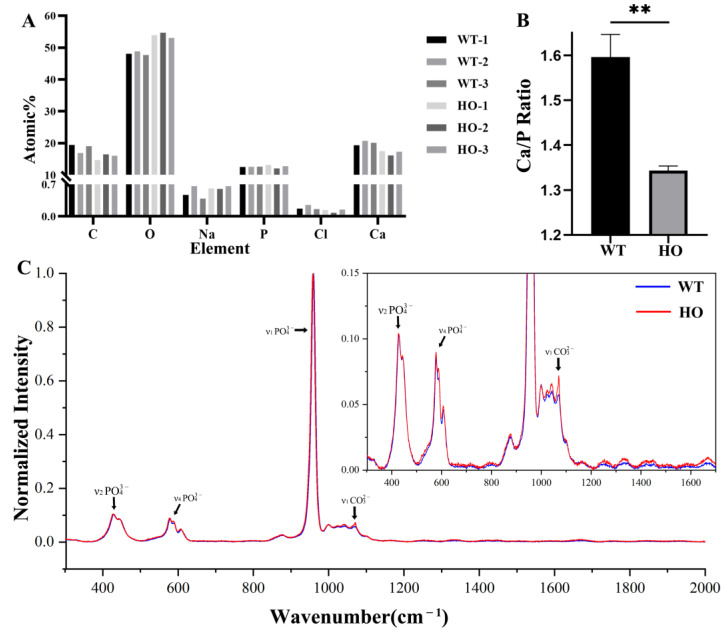
Enamel mineral composition and crystal properties: (**A**) Element analysis data of the WT and HO mice enamel by atom% from EDX. (**B**) The average Ca/P ratio of enamel samples in HO mice was significantly lower than that in WT mice: ** *p* < 0.01. Error bars denote mean ± SD. (**C**) Overlapping normalized spectra of WT mice enamel (blue) and HO mice enamel (red).

Energy dispersive X-ray spectroscopy (EDX) mapping for C, O, Na, P, Cl and Ca contents by atom% of middle layer enamel in each sample are displayed in the statistical chart (Figure 6A). EDX disclosed Ca/P = 1.60 ± 0.05 (atomic %) for normal mice enamel and Ca/P = 1.34 ± 0.01 (atomic %) for HO mice enamel. Crystallographically, the enamel of HO mice showed calcium deficiency compared to the enamel of WT mice (Figure 6B).

In the test, the position of the bands was nearly the same in the WT and HO mice enamel as reported previously (Figure 6C), demonstrating that the major mineral constituents did not vary strongly between the WT and HO samples. However, relative to the main phosphate vibration at 959 cm^−1^, several changes could be observed in the HO mice enamel, for example, the ν_1_ CO_3_^2−^, because of the type-B carbonate substitution in the apatite lattice in the defective enamel. The ν_1_ PO_4_^3−^ band associated with the P-O stretch shifted a little from 959.35 ± 0.10 cm^−1^ in the WT samples, and to 959.02 ± 0.10 cm^−1^ in the HO samples (Table 2). The FWHM of the ν_1_ PO_4_^3−^ band was found to be wider in the HO samples (15.11 ± 0.27 cm^−1^) than in the WT samples (13.83 ± 0.14 cm^−1^) (*p* < 0.001). Additionally, the carbonate-to-phosphate ratio, ν_1_ CO_3_^2−^/ν_1_ PO_4_^3−^ (relative integral areas), of the HO mice enamel was higher than in the WT mice enamel (*p* < 0.01).

## 4. Discussion

To the best of the authors’ knowledge, this is the first report of the *Kdf1* missense mutation knock-in mice model. In our study, we examined the enamel structure defects in the mice model. Specifically, abnormally fragile enamel prisms, reduced mineral density, altered crystallinity and chemical composition of the enamel layer were testified for the first time in the adult mice, all of which corroborated the intrinsic defects in their enamel. Based on our analysis and previous work, the *KDF1* gene is clearly a crucial gene associated with enamel development in humans and mice [19,21].

*KDF1* was first reported by Lee et al. [18]. They found *Kdf1* mutant mice displayed thickened epidermis and defective epidermal barrier formation as a result of keratinocyte defects by a *Kdf1* knockout model. Yet the function of *Kdf1* in tooth germ development cannot be determined because of postnatal death. It is worth noting that their knockout mouse served as a loss-of-function model whose phenotype should be different from our missense mutation model. We later found *KDF1* mutation (c. 908G>C, p.R303P) affects tooth development in patients, and *Kdf1* expression is essential for the development of teeth [21]. In the present study, we further show that teeth from the *Kdf1* missense mutation (c. 908G>C, p.R303P) knock-in mice model displayed a flawed and defective structure (Figure 3). Specifically, their enamel prisms were not only shaped abnormally with a loose, decussating crystal arrangement, but part of them fused and collapsed, leaving large gaps. Furthermore, misshapen crystals were disorderly combined within each prism (Figure 4). Crystallinity and chemical composition in the enamel hydroxyapatite were also affected by mutation (Figure 6, Table 2). These results indicated that *Kdf1* plays an important role in mouse enamel development.

Before our study, several pieces of clinical evidence supported the critical role of *KDF1* in the number of teeth and enamel formation [16,19,20]. However, previous studies were all related to the genetic analysis of patients and lacked animal models which may provide invaluable information. In this study, we first generated a *Kdf1* missense mutation (c. 908G>C, p.R303P) knock-in mice model in which we observed the specific effect of *Kdf1* gene mutation on the tooth by characterizing their morphological, crystallographic and chemical characterizations of enamel through combined spectroscopic techniques.

Interestingly, compared to human patients, the *Kdf1* missense mutation (c. 908G>C, p.R303P) knock-in mice model showed vital but distinctive dental manifestations. Several reasons we speculate are as follows: First, patients with *KDF1* missense mutation showed non-syndromic, missing and malformed teeth. At the same time, their enamel was not mineralized well with a chalky, flat and caries-active appearance in their deciduous molars and the enamel layer seemed radiolucent in the panoramic photographs [21,22]. Additionally, the different genetic background of humans and mice sometimes affect their dental phenotype. For example, Courtois [30] found tooth agenesis and conical teeth are associated with a heterozygous missense mutation at serine 32 of *IkappaBalpha(lkBa)* in humans. However, mice expressing a transdominant negative mutant of lkBa had third molar agenesis, with other molars severely flattened [31]. In general, the hypohidrotic ectodermal dysplasia tooth phenotype appears to be less severe in mice than in humans, which may imply that species-specific differences in pathway utilization exist and/or compensatory pathways may be absent in humans [32].

To our knowledge, composition, as well as misorientation of HAP, is particularly critical for enamel to build up unique resilience and wear resistance [1,2,4,33]. Otherwise, unregular patterns easily lead to enamel defects [1,34]. The phenotype of maldeveloped enamel caused by genetic mutations can display a totally disordered arrangement of prisms in the enamel layer [14,23,25,35,36]. For example, the mutation in the *MMP20* or *KLK4* gene in mice resulted in grossly malformed enamel where the rod’s pattern was unrecognizable and the interprismatic enamel was almost absent. Their composing enamel rods are prone to stress during mastication [10,11,12,13,14,15]. In our study, we could see the loosely packed enamel rods as well as those wider sheath regions in the HO mice enamel layer. Additionally, a collapse at the tip of the cone-shaped enamel rods was confirmed by both SEM and AFM. Thus, it may be hypothesized that the less dense nature combined with the wider interprisms and the deformed enamel rods may allow shearing forces to cause fracture and loss of tissue [5]. The observed defective appearance and reduction in Young’s modulus were therefore closely associated with the microstructural disorder. A further aspect to consider is that the enamel defects could be affected by a diet that produces wear when chewing [37]. However, it is unclear if the defective structure was caused by wear or simply by the tooth’s flawed biomechanics, in which the enamel was unable to sustain bending stress during mastication and chipped off, exposing the underlying dentin.

The Raman spectrum of enamel is highly correlated with the most dominant phosphate (ν_1_ PO_4_^3−^) groups, which we used to analyze the regularity of enamel crystals. Other bands related to the ν_2_ and ν_4_ PO_4_^3−^ vibrations are detected between 390 cm^−1^ and 490 cm^−1^ and 560 cm^−1^ and 625 cm^−1^, respectively; this is consistent with previous studies [38]. FWHM can indicate the uniformity of crystal alignment [39]. Widened FWHM of ν_1_ PO_4_^3−^ band at 959 cm^−1^ in HO samples suggested poor alignment and uneven quality of HO mice enamel. Because of the overall similarity in the Raman spectra of all samples, the major end-products are not expected to be too different. They still maintained a relatively well-resolved band structure. However, the presence of crystalline phosphate-based minerals in HO mice enamel may not be as uniform as those in natural enamel minerals. Generally, carbonate reduces the crystallinity of apatite and alters its shape so that crystallites are rather equiaxed than resemble needles [40]. Data suggested that the HO mice enamel contained a higher carbonate content (relative to phosphate) than the WT mice enamel. Due to the inverse relationship between mineral crystallinity suggested by the phosphate band and the degree of carbonation, it is plausible that the higher carbonate concentration affected the crystallinity of the HO mice enamel.

The results of our study have confirmed enamel defects in adult *Kdf1* missense mutation (c. 908G>C, p.R303P) knock-in mice. However, there are some limitations to this study. Firstly, our study generated most results from adult mice. More information on the morphological structure and mechanical properties of tooth enamel from younger mice need to be provided. Additionally, more details on cellular characteristics of tooth development are needed to explain how the altered gene affects the enamel structure. Investigations of the alterations on ameloblast membrane integrity are underway in our laboratory.

## 5. Conclusions

In this study, we revealed an alteration of the enamel structure in the novel *Kdf1* missense mutation knock-in mice model, including incomplete appearance, declined volume, the mineral density of the enamel layer, irregular gathering of nanofibrous apatites and changed mineral composition, which suggested that *Kdf1* mutation might lead to enamel structure disorder. Combined with our previous results, *KDF1* may contribute to the enamel formation.

## Figures and Tables

**Table 1:** The average surface roughness (Ra), root mean squared surface roughness (Rq), maximum surface roughness (Rmax) and Young’s modulus of the middle layer of the enamel.

Parameters	WT	HO	Statistically Significant Difference
Ra (nm)	20.23 ± 8.39	82.90 ± 20.72	<0.01 **
Rq (nm)	23.57 ± 5.54	98.43 ± 14.28	<0.01 **
Rmax (nm)	195.0 ± 34.83	826.73 ± 199.12	<0.01 **
Young’s Modulus (Gpa)	75.57 ± 6.96	54.89 ± 3.70	<0.05 *

* Values represent the mean ± SD. Significant difference from the HO mice enamel. * *p* ≤ 0.05, ** *p* ≤ 0.01.

**Table 2:** Descriptive statistics of the distribution of the normalized Raman parameters of the WT and HO mice enamel.

Parameters	WT Mice Enamel		HO Mice Enamel		Statistically Significant Difference
	Average	SD	Average	SD	
Position of the Phospate ν_1_ band	959.35	0.10	959.02	0.10	n.s.
FWHM of the Phospate ν_1_ band	13.83	0.14	15.11	0.27	<0.001 ***
Position of the Carbonate band	1070.92	0.38	1070.67	1.07	n.s.
FWHM of the Carbonate band	16.71	2.11	17.04	3.47	n.s.
Ratio of Carbonate/Phosphate	0.034	0.02	0.047	0.05	<0.01 **

Significant difference from the HO mice enamel, *p* ≤ 0.05. ** *p* ≤ 0.01, *** *p* ≤ 0.001.

## Data Availability

The data are available from the first author.
